# Oral and emotional health experience of refugees’ in the state of Massachusetts - A mixed methods approach

**DOI:** 10.1371/journal.pone.0281361

**Published:** 2023-03-09

**Authors:** Shaikha Aldukhail, Anubhuti Shukla, Mohammad Tareq Khadra, Ziad Al Hennawi, Samantha Jordan, Tamara J. Cadet, Hend Alqaderi

**Affiliations:** 1 Department of Preventive Dental Sciences, College of Dentistry, Princess Nourah Bint Abdulrahman University, Riyadh, Saudi Arabia; 2 Department of Oral Health Policy & Epidemiology, Harvard School of Dental Medicine, Boston, MA, United States of America; 3 Department of Cariology, Operative Dentistry and Dental Public Health, Indiana University School of Dentistry, Indianapolis, IN, United States of America; 4 University of Southern California, Los Angeles, CA, United States of America; 5 Edward M. Kennedy Community Health Center, Worcester, MA, United States of America; 6 Lowell Community Health Center, Lowell, MA, United States of America; 7 Simmons School of Social Work, Boston, MA, United States of America; 8 Dasman Diabetes Institute, Kuwait City, Kuwait; International Medical University, MALAYSIA

## Abstract

**Objectives:**

In this study, we aimed to explore the oral and emotional health challenges experienced by a sample of refugees in Massachusetts across different stages of resettlement using a mixed methods approach.

**Methods:**

We collaborated with two Federally Qualified Health Centers to identify and recruit participants for either surveys (n = 69) or semi-structured interviews (n = 12). Data collection was conducted in 2018. We performed descriptive statistics using STATA 14, and analyzed the interviews using qualitative methods.

**Results:**

Overall, cost and lack of structure were the largest barriers identified for accessing dental care in participants’ home and host countries. In the US, participants reported receiving state-provided public health insurance, but still experienced disrupted access to dental care due to coverage limitations. We identified several mental health risk factors that may affect participants’ oral health, including trauma, depression, and sleeping problems. Despite these challenges, participants also identified areas of resilience and adaptability in both attitude and actions.

**Conclusions:**

The themes identified in our study suggest that refugees have attitudes, beliefs, and experiences that contribute to their perspectives on oral health care. While some of the reported barriers to access dental care were attitudinal, others were structural. Access to dental care in the US was reported to be structured and available, but with limited coverage issues. This paper underscores the oral and emotional health aspects of refugees for future considerations and planning of appropriate, affordable and cost-effective policies in the global health care systems.

## Introduction

The current trend of high refugee and displaced populations is considered one of the largest global challenges since World War II. The United Nations Commissioner for Refugees (UNHCR) estimated that in 2020, about 82.4 million individuals were forcibly displaced as a result of persecution, conflict, generalized violence, or human rights violations [1]. A key aspect related to displacement is that of health. The relationship between displacement and health is complex and dynamic, Which requires host countries to develop robust healthcare systems that can handle the growing demand for healthcare among these migrant populations [2]. Additionally, health is strongly related to social determinants and is often not equally distributed among different population groups, leading the migration process to expose the most vulnerable socioeconomic subgroups to a myriad of problems [2, 3]. Not only are many refugees contracting diseases during their displacement and upon arrival in host countries, they are also being exposed to trauma and high levels of stressors, which can affect their mental and emotional wellbeing [3, 4].

Many studies have documented a link between oral health and mental health, but none have specifically examined this relationship among refugees [5–8]. Oral health deserves special attention in refugee populations in the United States, as it is well established that proper oral health is essential for daily activities such as talking, eating, smiling, sleeping, and their self-esteem, all of which are determinants of overall health and well-being [9–11]. Additionally, many reports have highlighted the link between oral and physical health, suggesting that poor oral health may lead to poor physical health [10, 12–14]. However, oral health is not often a priority when it comes to identifying and treating health needs during times of conflicts and war. One possible explanation for this lack of attention to oral health may be due to that refugees focus on urgent medical care and basic needs, such as access to drinking water, a nutritious diet, and other sanitary conditions [15]. Improving access to oral health care and ultimately, the oral health status of refugees’ is a global priority.

Compared to vulnerable populations in host countries, refugees’ reported experiencing higher prevalence of oral diseases and barriers to access to dental care [16]. Oral disease is the most common problem among refugee children and the second most common problem among refugee adults [17]. Upon their arrival to the United States, refugees do get screened for dental diseases as part of the medical screenings and may also receive state funded public medical and dental insurance. However, limited coverage and lack of a dental home makes it difficult for this population to access oral health services consistently [18]. Recent recommendations by the World Dental Federation (FDI) emphasized the fact that oral health is key to prevention and control of Non-Communicable Diseases (NCDs). The current momentum for NCDs presents an opportunity to improve oral health on a global scale through continued advocacy for the integration of oral diseases into action plans for prevention and control of NCDs [19]. Healthcare organizations in host and resettlement countries should quickly adapt and reform to meet the changing needs of this growing patient population. Therefore, research in this area is needed to learn about the disease experience and patterns of refugees who became permanent residents in the United States, with a focus on the current needs for optimal oral and emotional health. To the authors’ knowledge, there are no investigations describing the oral and emotional health of the refugee population in the United States.

The aims of this study is to provide a deeper understanding of the oral and emotional health challenges experienced by a sample of refugees in the state of Massachusetts, both before and during their fleeing period, and after arriving to the United States. Using a mixed method approach, the study has three main objectives: 1) To qualitatively describe the oral and emotional health challenges experienced by the sample of refugees across different stages of resettlement. 2) To quantitatively describe the status of oral and emotional health for refugees in Massachusetts. and 3) To assess the access to dental care for refugees after arriving in the United States for future needs assessment.

## Methods

This study was conducted in collaboration between Harvard School of Dental Medicine and two Federally Qualified Health Centers (FQHC) in the State of Massachusetts. The refugees list was provided by the Health Centers’ administrations. Participants were recruited by inviting them through mail and then following up with a phone call to schedule appointments, with a second call as a reminder two days before their scheduled visit. Appointments were held at both FQHC. Before data collection, participants were given enough time to read and sign a written informed consent form available in either Arabic or English. Children were given a child assent form and a consent from to their parents to sign. Participants were given a copy of the consent information.

A mixed method approach, combining both qualitative and quantitative methods, was used to provide context, via the in-depth, open-ended investigation, of matters that cannot be quantified or broken down into defined choices per se. The participants’ sample for the quantitative and qualitative study were different and exclusive of each other.

### Qualitative methods

To obtain participants’ personal perspectives on their oral and emotional health experiences across different stages of resettlement, we designed a qualitative semi‐structured interview protocol to answer the research question: “What are the dental and emotional health experiences of refugees in one city at the state of Massachusetts across different stages of resettlement?”.

We recruited 12 informants representing countries from Syria, Iraq, Sudan, Afghanistan and Zimbabwe using convenient sampling. Each interview lasted approximately 45 minutes to 1 hour and focused on the participants’ personal perspectives on their oral and emotional health experiences. In order to assess refugees’ emotional health we used an open-ended modification of the validated adult behavioral check-list which indicates risk factors of mental health [20].

### Procedures

Two investigators conducted individual interviews in a private exam room at the FQHC. While (SA) was interviewing the participant, (HA) took field notes for data enhancement to provide additional context for analysis. Additionally, SA kept a research journal as evidence of the interview process and enable reflection on personal roles and biases that might have influenced the analysis [21]. The journal was shared with (TC) to ensure reflexivity, a crucial component of phenomenology. The interviews were conducted in a natural conversations manner to evoke authenticity and develop rapport and establish a safe space for participants to share their personal narratives and experiences. Each overarching question was followed up with more specific questions and probes as appropriate [22]. Interview questions explored participants’ past history in their home country including access to oral health care and related issues, and some of the possible barriers to access oral health in the United States, oral health preventative behaviors, social and emotional behaviors. The interviews were recorded using a tape‐recorder and later uploaded to a password protected computer via a secure server at Harvard University School of Dental Medicine (HSDM) using a virtual private network. Each interview was transcribed and translated verbatim to English by the first author SA, and all identifying information was removed before this process.

### Quantitative methods

We recruited a convenient sample (n = 69) participant from multiple countries: 47 adults and 22 children. At each designated study site, an assigned investigator met with the participant, explained the study, before administering the questionnaire. Participants were asked if they experienced one or more of the specific dental/oral problems in the past month (e.g. bleeding gums when brushing teeth or pain in teeth while consuming hot or cold food or drink). Additional survey questions determined the individual’s perceived need for dental care (‘‘Do you currently have any cavities in your teeth?”; “Do you currently have painful gums or gums that bleed easily?”), reasons why they may not receive regular dental care. The questionnaire was administered in paper in presence of the parent/ guardian when applicable. If the participant did not have a regular dentist, they were given a list of dentists near their residence and information on accessing those services. Toothbrush and dental floss were provided to each participant in addition to oral health instructions.

### Inclusion criteria

To be eligible for participation in this study, participant needed to: 1) be older than 11 years of age; 2) have resettled to the United States within 3 years before data collection in 2018 i.e (2015–2017); and 3) be of a refugee status who spoke either Arabic or English. Ethical approval for this research was obtained from the Harvard Human Protection Program, Harvard Faculty of Medicine, Office of Human Research Administration (Protocol #IRB17-077).

### Quantitative analysis

A single research assistant entered all clinical data directly into a password-protected database. Descriptive statistics were performed using STATA 14. Frequency analysis of various variables were obtained using cross tabulations. Proportions and numbers were used to describe the categorical variables stratified by adults and children.

### Qualitative analysis

We employed well-documented qualitative analysis techniques by sorting the data into themes that emerged from responses to our open-ended questions [23, 24]. Two coders SA and TC, separately examined the first three transcripts to identify possible codes. Then they met regularly to create a codebook and went back to code the remaining transcripts using holistic reading and selective coding approaches. The transcripts were re-coded by SA using the final codes from the codebook and reviewed by TC to ensure trustworthiness in the coding process [25, 26]. Any discrepancies in coding were resolved through consensus between the two coders. Through ongoing comparison, we were able to place emerging themes and subthemes in relation to each other, and we reached data saturation when no new themes emerged [24, 26]. Following the common approach of using codes from a codebook to tag segments of text and sort text segments with similar content into separate categories, we distillation the data into three overarching themes [22, 24, 26]. The most revealing themes came from the asking: “What statements or phrases seem particularly essential or revealing about the experience being explored?” Fifteen emerging themes were compressed into three overarching themes which include: 1) Barriers to access to dental care in home country, during migration/resettlement and post-settlement 2) Emotional health due to trauma; and 3) Resiliency characteristics.

## Results

### Quantitative study results

#### Participant characteristics

Majority of adult participants were between 25–44 years of age while most of the children were 11–13 years of age. Most were from Syria (adults 60% and children 40.9%). Relevant descriptive information of the survey respondents is represented in **(Table 1)**.

**Table 1:** Participants demographic characteristics.

**Characteristics**	**Qualitative interviews participant Characteristics (n = 12)**			
	**% (n)**			
**Sex**	Male	58% (7)		
	Female	41.17% (5)		
**Education level**	Elementary school	16.67% (2)		
	Some High school	25% (3)		
	High school	25% (3)		
	Bachelors	33.33% (4)		
**Marital status**	Single	25% (3)		
	Married	66.67% (8)		
	Divorced	8.33% (1)		
**Employment status**	Work at a job	33.33% (4)		
	Don’t work at a job	50% (6)		
	Student	16.67% (2)		
**Characteristics**	**Quantitative Survey participant Characteristics (n = 69)**			
	**Adults (n = 46)**		**Children (n = 22)**	
	**% (n)**		**% (n)**	
**Age**	18–24	17% (8)	11–13	54.5% (12)
	25–44	45% (21)	14–16	36.4% (8)
	45–64	36.2% (17)	17–18	9.1% (2)
	65+	1.8% (1)	-	-
**Sex**	Males	51.1% (24)	Males	63.6% (14)
	Females	49.9% (23)	Females	35.4% (8)
Nationality/origin*	Syrian	60% (27)	Iraqi	31.8% (7)
	Iraq	31.1% (14)	Syrian	40.9% (9)
	Afghanistan	8.9% (4)		
**Marital Status**	Married	71% (33)		
	Single	23%(11)		
	Other	6% (3)		
**Employment status**	Work at a job	35% (16)		
	Don’t work at a job	38%(19)		
	Retired/other	27%(12)		
**Education levels**	Bachelor’s	30% (14)		
	High school	36% (17)		
	Less than high school	13% (6)		
	No schooling	21% (10)		

#### Participant oral health characteristics

Over half of the participants in both adult (55%) and children (52.4%) groups brushed their teeth twice a day. About half of the adults flossed regularly (54%). The majority of the participants among both children (72.7%) and adults (78.2%) reported having visited a dentist in the past six months **(Table 2)**. Most participants did not report having any difficulties in scheduling dental appointments, but those who did, cited the most common barrier being language issues (32.6%).

**Table 2:** Participants’ oral health characteristics.

**Qualitative interview participants**		
**Last Dental visit**		**% (n)**
visited a dentist less than 6 months ago		75% (9)
**Reason for last visit**		
Check up and cleaning		66.67% (8)
Emergency /pain		16.67% (2)
**Experienced dental pain in the past 12 months**		50% (6)
**Experienced painful and/or bleeding gums in the past 12 months**		50% (6)
**Was ever self-conscious because of their teeth**		41.17% (5)
**Quantitative Survey participants**		
	**Adults**	**Children**
**Last Dental visit**	**% (n)**	**% (n)**
Last visited a dentist less than 6 months ago	78.2% (36)	72.7% (16)
**Most common reason for last visit**	Restorations/fillings 26% (12)	Check up and cleaning 32% (7)
**Experienced dental pain in the past 12 months**	39% (19)	43% (10)
**Experienced painful and/or bleeding gums in the past 12 months**	39% (19)	n/a
**Oral Hygiene practices**		
Brushing twice a day	55% (17)	52.4% (11)
Flossing	45% (14)	n/a
Restricted Sugary drinks to special occasions	32% (14)	62% (13)

#### Emotional health challenges due to trauma

**Fig 1:**
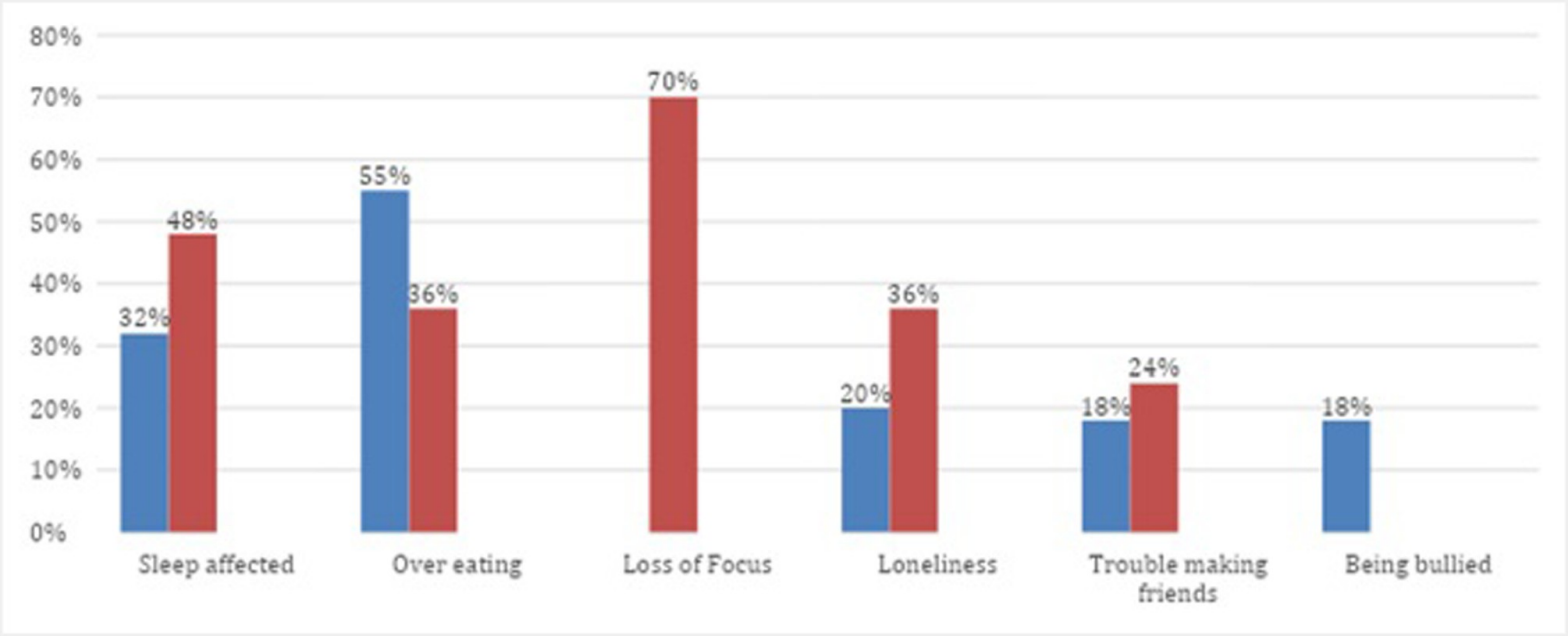
Refugees emotional health challenges among children and adults.

Majority of adults reported having loss of focus (70%). Being bullied was common among some children (18%) and the majority of them often also resorted to overeating (Fig 1).

### Qualitative study results

#### Participant characteristics

The twelve informants (n = 7 men, n = 5 women) between 21 and 66 years of age. The participants were from Syria, Iraq, Sudan, Afghanistan and Zimbabwe (**Table 1**). The majority visited a dentist within the past six months (75%). Half of the informants have experiences oral pain is the past year (50%), while (41.17%) reported feeling self-conscious because of their teeth (**Table 2**).

To contextualize the data analysis, refugees’ narratives were used. However, due to the sociopolitical climate surrounding refugee resettlement, no city name or interviewee names were used to preserve the confidentiality of our participants. While no names were used, we have not altered any other information (e.g. specific narratives, events and experiences). Findings included the following three themes: 1) barriers to access to dental care in home country, migration/resettlement and post-settlement; 2) Risk factors to mental health due to trauma; and 3) resiliency characteristics.

#### Barriers to access dental care in home country, migration/resettlement and post-settlement

Overall, cost was the largest barrier associated with accessing dental care in participants’ home country **(Table 3)**. Their experiences were slightly different and better during the migration/re-settlement period in host countries with participants able to access care. In some cases, participants were able to access care with a provider from their home country. In the US, while all participants received the states’ public insurance (Medicaid), they still reported disrupted access to dental care because of dental coverage limitations. Finally, despite the barriers described by participants, they did communicate their ability to successfully schedule dental visits. See **(Table 3)** for an overview of their oral health characteristics after settling in the United states.

**Table 3:** Qualitative interview findings “participants quotes”.

Main themes	Sub themes	Participants Quotes
A) Barriers to access to dental care		
**1- In home country**	**Cost**	• *” Dental care was expensive and almost all dental care was private in Zimbabwe*.*”* • *“(referring to Iraq) We would go get private treatment*. *Dental care was somewhat expensive*.*”“And the public clinics had long lines*, *and I was too scared to go and wait for long*.*”* • *“(referring to Syria)*, *Dental care can be bought with money*, *nothing is free”*
**2- Migration/resettlement**	**Access to care**	• *“…a dentist who was from my mom’s village (referring to Syria) opened a clinic in Turkey*. *He treated me for free*. *Whenever we needed dental treatments*, *we went to him and he treated us for free or for a small fee*.*”* • *“We found an affordable Syrian dentist that we went to and he treated me and my husband*. *(referring to Lebanon)”* • *“Because we had a UN refugee warrant*, *we had the opportunity to be treated for free in governmental Dental clinics at Jordan*. *In these*, *you could extract and clean teeth for free*, *but fillings and root canals were not free”*
**3- post-settlement in USA**	**Medicaid coverage limitations**	• *“…I had to change clinics a lot*, *because every time I go to a new one*, *they tell me that your insurance doesn’t cover the required treatment”* • *“I have changed clinics 4 times that’s why (referring to insurance)*. *They extracted 2 of my teeth because our insurance doesn’t cover root canals*. *They asked me to pay and when I told them that I can’t they took my teeth out*.*”*
	**Scheduling Dental visits**	• *“No not at all(referring to difficulty in scheduling an appointment)*.*They sometimes give you an appointment within a month or 15 days” “Once my husband was in pain and called for an appointment and they gave him one in 2 weeks*, *but when he said that it was an emergency they immediately gave him one the next day*.*”* • *“They called every 6 months*, *to do an exam*, *a cleaning and take x-rays*.*”* • *“Someone from the international institute came to our home after moving to Massachusetts and told us that we need to visit the dentist*, *and he helped us in making an appointment*.*”*
**B) Risk factors to mental health**	**Trauma**	• *“I was an engineer and worked in the American camp in Afghanistan*. *My daughter -also an engineer- worked with me*. *We received death threats -to kill us both- for working with the Americans so we had to leave*.*”* • *“On 3 different occasions our home got bombed and collapsed right on top of our heads*. *(Referring to Iraq)”* • *“Back in 2011 I was captured for 28 days*. *I have no idea why and how I got out of that situation*. *I will need to see 50 psychologists after all the torture I have experienced in that Damascus prison*.*”*
	**Other Emotional health issues due to trauma:** (sleeping problems, depression risk factors, social isolation)	• *“I am tired from always thinking about my family in Syria and Turkey*. *Sometimes*, *I feel so helpless that all I to do is close my eyes and avoid interacting with anyone … back [home] I used to work more- I cleaned*, *washed*, *and lifted heavy objects all day*. *But now I don’t have the energy anymore–I am always tired and lazy*.*”* • *“I am not satisfied with my work performance*. *I think my performance is poor*. *I would like to give more and I am able to give more in my job*. *But I have no energy; I am lazy*.*”* • *“I also suffer from depression and psychological problems- I have been looking for a therapist for 3 years*. *But no one was referring me*, *they just gave sleeping and sedative pills…I’ve always felt like my body was tired and that I couldn’t bring myself to do anything–Sometimes I can’t even cook or clean*. *I feel sad and I’m always crying*. *Oh I cry a lot…”* • *“Some days when I’m feeling low*, *I can’t bring myself to do anything around the house*. *I sleep most of that time*.*”* • *“I don’t sleep well*. *My sleep gets interrupted a lot*, *I keep sleeping and waking up*. *I do have nightmares*. *Some are really frightening*. *I guess they happen because of how much I think about my family’s and son’s safety*.*”* • *“I don’t sleep well*. *I sleep too little*. *I also have a lot of nightmares [and] this started when that incident (referring to trauma) happened*.*”*
**C) Resiliency characteristics**	**Recognizing mental health concerns and help seeking characteristics**	• *“I see a therapist for my issue and talking to him helps a little…”* • *“Yes*, *I always feel tired without a reason*, *and I have a therapist and I have a psychiatrist who prescribed me medication*. *I am doing better now*.*”* • *“Oh yes I do feel tired and low without a medical reason*, *but I didn’t see a doctor for this because I do not have a physical problem*.*”*
	**Family well-being**	• *“I feel safe here and I don’t have to worry about my family’s safety*.*”* • *“Thank god my kids are safe*, *and we are all healthy—when you see how others around the world are suffering your own problems seems small*.*”* • *“I came to America for my boys*. *I lost my house*, *my job*, *my social status and everything by coming here and starting all over*. *There is a difference between when I was a Doctor and now in being an unemployed person who receives aid from the government*. *My priority as a mother is to make sacrifices so my children can live in peace and have a better future*.
	**Social integration characteristics**	• *“I get along with others easily*. *I love to socialize and interact with people*. *Nothing makes me happier than interacting with people and forging relationships*.*”* • *“It’s not the same as back home*, *but I am getting along with other people*. *Not difficult at all (in response to difficulty to make friends in the US)- I am always trying to make new friends*.*”* • *“I am still optimistic*. *I made so many friends and will continue making new ones*.*”*

#### Emotional health challenges due to trauma

There were many mental health risk factors identified by participants that might affect oral health [5–7]. These concerns were related to trauma, sleeping problems and risk factors for depression. Much of the trauma experienced explained why participants relocated to the United States **(Table 3)**. These trauma experiences seem to be related to their past and current reports of feeling depressed, having sleeping problems, issues with friendships and social networks. For example, some participants commented on their fatigue and lack of energy and motivation **(Table 3)**. Their comments about sleeping problems were related to past traumatic experience and could be a possible risk factors for depression.

#### Resiliency characteristics

Despite the mental health challenges, participants identified a number of areas that can be considered to be resiliency and adaptability skills. These include: Participants’ awareness of risk factors for mental health problems, care about family well-being and interest to invest in friendships and social networks. While some participants recognized their mental health concerns and sought help, others recognized the need for help but did not see mental health as a physical problem and did not seek help **(Table 3)**. Family well-being was a characteristic that appeared to help participants make sense of their lives in their home country and adapting to living in the United States. Participants reported appreciation for the availability of healthcare resources including the FQHC, the state’s public health insurance and the different orientation and integration programs in the state of Massachusetts. They also had a positive outlook on their new life and eagerness to integrate and socialize despite the different environment.

## Discussion

This study aimed to provide a deeper understanding of the refugee experience related to dental and emotional health for a given sample of refugees’ in the state of Massachusetts, across the stages of resettlement. We explored access to dental care at their home country, during their fleeing period (i.e. host countries), and finally, after arriving to the United States. Our findings indicated that while there may have been barriers to access reliable dental care in the participants’ home countries and afterwards in host countries, this had improved as they moved to settle in the United States. Some previous barriers such as lack of structure were eliminated in the US. However, other barriers such as affordability and limited dental insurance coverage for services that are not preventative were reported as emerging issues in Massachusetts.

The qualitative analysis revealed that a high proportion of our sample had experienced dental pain, and/or bleeding gums. This clearly reflects a need for dental care, and it might be due to the inadequate coverage offered by the limited public insurance that is offered to this population. Only about half of the adult and children participants brushed their teeth daily which suggests inadequate health literacy and the need for dental health education.

The mental health risk factors due to trauma shared by the participants were just as important as their oral health needs. It is critical to consider these emotional health challenges individually but also within the context of oral health needs. It is worth noting that the majority of our participants did not stay in refugee camps before arriving in the United states, which suggests that they may have had a relatively higher socioeconomic status. This is evident by their ability to afford rent and dental treatment in host countries and successfully navigate the complex process of applying for asylum in the United States. Our participants received comprehensive dental treatments, such as prosthodontic and endodontic procedures, before arriving to the United States. However, the required maintenance services for these treatments were no longer covered by the public health insurance they received in Massachusetts. In comparison to host countries, these refugees reported receiving more systematic and structured services upon arrival in Massachusetts, such as orientation and integration programs aimed at finding employment and reducing reliance on cash assistance, in addition to the public health insurance that includes medical, mental, and dental care [27].

Growing evidence suggests an association between oral health and mental health, as they share common risk factors such as socio-economic status and lifestyle habits [5–8]. Our sample reported emotional distress and a risk of mental illness, such as loss of focus and poor sleep. As a result, it is crucial to address common risk factors in order to maintain optimal oral and overall health [13, 14]. Traditional oral health interventions have mainly focused on treating the dentition and the periodontium, with little attention given to the underlying social, behavioral and systematic factors. Therefor it is essential to shift this approach and encourage health care providers to consider the social and behavioral determinants of health and prioritize physical and social wellbeing.

Despite the trauma experienced of this sample of refugees, they demonstrated resilience and adaptability, which healthcare providers can use to support their dental and mental health needs. most of the participants expressed optimism despite their past and current experiences. While most reported feelings safe and secure after migration, they also acknowledged that the process of resettlement in the US was not easy, particularly due to post-migration stressors. Understanding factors related to resilience, such awareness of oral health needs, mental health risk factors, prioritizing family well-being and choice of social networks, may help mitigate the impact the impact of previous trauma in the post-migration context. This aligns with previous research that demonstrated that refugees’ mental health was greatly impacted by post-migration living conditions [28]. In addition, cultural barriers and potential internalized stigma, such as the participant who did not see a therapist because they did not view mental health problems as physical issues requiring medical attention, may affect help-seeking behaviors [29]. Self-stigma in relation to mental health is defined as *“the negative beliefs an individual holds about their own psychological symptoms or help-seeking”* [30].

Healthcare providers working with refugee populations must recognize the connection between oral health and mental health and the continued presence of health disparities for this vulnerable group [5–8]. Providers must take into account cultural and contextual factors while familiarizing themselves with the sociopolitical effects of displacement and resettlement in their interaction with this population. Cultural competency training for healthcare professionals dealing with refugee or migrants may be necessary to improve access to and utilization of dental services. Ignoring the social differences could discounts the fact that the roles of the researcher and participants always shape the interview process, and that the act of interviewing is invasive [31, 32]. Additionally, it is important to recognize some of the cultural nuances associated with working with the refugee population in a research setting. These factors were of the utmost importance for all authors’ particularly SA who shares similar cultural values as the participants in Qualitative interviews. This similarity may have made it easier for participants to share their experiences with her in the interview, in addition to the trust and rapport that was easily established.

Our study had some drawbacks as it was one of the first of its kinds. The cross-sectional nature of the surveys challenges our ability to examine causal relationships. The study relied on voluntary participation, which may have introduced selection bias, and social desirability bias may have affected the responses of participants. Additionally, even though thematic saturation was achieved in our interviews, our sample size, while appropriate for our exploratory purpose, could have been larger and more inclusive. Increasing the sample size would have enabled us to gain a broader representation of narratives based on key variables such as country of origin, sex, age, and education. These findings should be interpreted with caution and not generalized beyond their heuristic value. Moreover, given the rapidly shifting sociopolitical landscape in the United State and fear of unanticipated repercussions, participants were hesitant to answer our calls or participate in our study, and when they did, interviewees were wary of accidentally revealing information that could be used against them.

They were also wary of providing information that could be used against them, a concern consistent with the experiences of some refugees whose information was used against them by government-sponsored public and private services in their countries [32].

## Conclusions

The mixed methods approach in the present study provided a comprehensive understanding of the attitudes, beliefs, and experiences of the refugee population in regards to oral health care. While some of the reported barriers to access oral healthcare were based on attitudes, others were structural, such as a lack of financial means or resources. Access to dental care in the home country and intermediate host country was described as limited, primarily due to either lack of financial means or lack of structure. On the other hand, access to dental care in the United States was reported to be structured but with limited coverage issues. Participants reported that post-war trauma may pose a risk to their mental health, but they also showed resilience and gratitude for the services and safety provided to them in the United States.

Additionally, comprehending the interactions between various health factors is crucial for targeting common risk factors and optimize health outcomes for this vulnerable and high-risk population. Offering a wider range of definitive dental services to these populations might save time, energy and resources, preventing the need for more extensive treatments later on. The US must implement sustainable strategies to improve access to oral and mental health care for refugees and asylum seekers. Several future research directions could facilitate our understanding of refugees and migrants’ resettlement experiences. This paper highlights the importance of oral and mental health for refugees and serves as a call to action for designing cost-effective and accessible health policies in the future.

## Supporting information

S1 File Inclusivity in global research. (DOCX)

S1 Data (XLSX)
